# Eupalinolide O Induces Apoptosis in Human Triple-Negative Breast Cancer Cells via Modulating ROS Generation and Akt/p38 MAPK Signaling Pathway

**DOI:** 10.1155/2022/8802453

**Published:** 2022-09-21

**Authors:** Yaping Zhao, Liping Fu, Jinbai Chen, Junhao Zhou, Chongmei Tian, Daotang Zhou, Rui Zhu

**Affiliations:** ^1^Department of Pharmacy, Shaoxing Hospital of Traditional Chinese Medicine Affiliated to Zhejiang Chinese Medical University, Shaoxing, Zhejiang 312000, China; ^2^Department of Pharmacy, The First People's Hospital of Kaili, Guizhou Province, Kaili, Guizhou 556000, China; ^3^School of Pharmaceutical Sciences, Zhejiang Chinese Medical University, Hangzhou, Zhejiang 310053, China; ^4^Department of Pharmacology, Nanjing University of Chinese Medicine, Nanjing, Jiangsu 210023, China

## Abstract

**Background:**

Triple-negative breast cancer (TNBC) is a subtype of breast cancer with limited therapeutic options. Eupalinolide O (EO) was reported to inhibit tumor growth. This study is aimed at exploring the role of EO on TNBC both *in vivo* and *in vitro. Methods*. In *in vitro* experiments, 3-(4,5-dimethylthiazol-2-yl)-2,5-diphenyltetrazolium bromide (MTT) and clonogenic assay were conducted to measure the impact of EO on TNBC cell growth at different concentrations and time points. Flow cytometry was conducted to evaluate cell apoptosis. Mitochondrial membrane potential (MMP) loss, caspase-3 activity, and reactive oxygen species (ROS) generation were assessed. The expressions of apoptosis-related mRNAs and Akt/p38 MAPK signaling pathway-related proteins were measured. In *in vivo* experiments, by injecting TNBC cells into the nude mice to induce xenograft tumor, mice were treated with EO for 20 days. Then, *in vivo* bioluminescence imaging system was utilized to monitor the growth and distribution of TNBC cells. Tumor volume and weight were also recorded. Hematoxylin-eosin (HE) staining and ELISA assay were applied to observe tumor tissue morphology and ROS levels. Furthermore, western blotting was conducted to observe the expression of apoptosis-related proteins and Akt/p38 MAPK signaling pathway-associated proteins.

**Results:**

EO inhibited the cell viability and proliferation of TNBC cells but not normal epithelial cells. Furthermore, EO induced apoptosis, decreased MMP, and elevated caspase-3 activity and ROS content in TNBC cells. Meanwhile, the expression of apoptosis-related mRNAs and Akt/p38 MAPK pathway-related proteins was regulated by EO treatment. Besides, *in vivo* experiments demonstrated EO not only suppressed tumor growth, Ki67 expression, ROS generation, and Akt phosphorylation but also upregulated caspase-3 expression and p-38 phosphorylation.

**Conclusion:**

EO may induce cell apoptosis in TNBC via regulating ROS generation and Akt/p38 MAPK pathway, indicating EO may be a candidate drug for TNBC.

## 1. Introduction

Breast cancer is one of the most commonly diagnosed cancers in females, and its incidence exceeds lung cancer. In 2020, there were about 2.3 million breast cancer cases and 0.6 million deaths from breast cancer worldwide [1]. Triple-negative breast cancer (TNBC) is a heterogeneous subtype of breast cancer, which accounts for 15-20% of total breast cancer [2]. In general, treatment of breast cancer typically differs by subtype [3]. Owing to the lack of amplification for the gene coding of the epidermal growth factor receptor 2 protein as well as the absence of hormone receptor expression, there are very limited therapeutic options for patients with TNBC [4]. At present, treatments for TNBC are still dominated by chemotherapy, but nearly all routine drugs will lead to drug resistance and have severe toxic side effects [5]. Therefore, it is an urgent need to investigate and develop new agents for the treatment of TNBC.

Apoptosis, a kind of programmed cell death, is inversely related to cell growth [6]. It is well established that induction of tumor cell apoptosis is an effective method for eradicating cancers [7]. Recently, accumulating evidence has shown that some proteins participated in antagonizing apoptosis, such as Bcl-2, are overexpressed in TNBC cells [8]. At the same time, several proteins that serve a proapoptotic function, such as Bax, are downregulated in TNBC [9]. In addition, the generation of reactive oxygen species (ROS) and the expression of Akt/p38 MAPK signaling pathway-related proteins are abnormal in most TNBC cells [10, 11]. Interestingly, the anti-TNBC effect of some anti-HER2 agents, such as neratinib, is accomplished by inducing the apoptosis of TNBC cells [12]. Hence, finding an agent that can induce TNBC cell apoptosis is an attractive strategy for the treatment of TNBC.

*Eupatorium lindleyanum* DC., a traditional Chinese medicine, is commonly used in the treatment of bronchopneumonia and influenza [13]. Recently, this herb has attracted more and more attention, because it was found that numerous compounds in the herb, such as Eupalinolide J and F1012-2, have been demonstrated to possess ability against TNBC both *in vivo* and *in vitro* [3, 14]. Eupalinolide O (EO), a kind of novel sesquiterpene lactone extracted from *Eupatorium lindleyanum* DC., has been proven to induce cell cycle arrest as well as apoptosis in human breast cancer cells [15]. However, the information on the effect and mechanism of EO on TNBC is limited.

In this research, we conducted both *in vitro* and *in vivo* experiments to explore whether EO can impede the development of TNBC by induction of apoptosis mediated by ROS generation and the Akt/p38 MAPK pathway.

## 2. Material and Methods

### 2.1. Cell Culture

Human normal breast epithelial cell line MCF 10A and TNBC cell lines (MDA-MB-231 cells and MDA-MB-453 cells) were gifted by iCell Bioscience Inc (Shanghai, China). All cells were cultured in Dulbecco's modified Eagle's medium (DMEM, SH30243.01, Hyclone, Utah, USA) with the addition of 5% fetal bovine serum (FBS, 11011-8615, Hangzhou Tianhang Biotechnology Co., Ltd., Hangzhou, China), 100 U/mL penicillin, and 100 *μ*g/mL streptomycin in a humidified incubator containing 5% CO_2_ at 37°C.

### 2.2. Cell Viability Assay

For evaluating cell viability, 3-(4,5-dimethylthiazol-2-yl)-2,5-diphenyltetrazolium bromide (MTT) assay was performed based on the manufacturer's protocol. In short, cells were plated at a density of 2 × 10^3^ cells/well into 96-well plates and then cultured with different concentrations (1-20 *μ*M) of EO [15]. EO was isolated from *Eupatorium lindleyanum* DC. by our research group and the purity was assessed by high-performance liquid chromatography (HPLC). The group treated with 0 *μ*M of EO was used as the control group. At the defined time points, each well was exposed to MTT solution (10 *μ*L/well, E606334-0500, BBI Life Sciences, Shanghai, China) for 4 h at room temperature (RT). After that, formazan crystals were dissolved by dimethylsulfoxide (DMSO) (100 *μ*L/well). Finally, the absorbance value was detected by a microplate reader (CMaxPlus, Molecular Devices, California, USA) at 450 nm.

### 2.3. Colony Formation Assay

To investigate cell proliferation capacity, clonogenic assay was carried out. Firstly, cells were plated in 6-well plates with 500 cells/well. Subsequently, the medium treated with 0-20 *μ*M of EO was changed at an interval of 3 days. Upon 2 weeks of incubation, the cells were rinsed by phosphate-buffered saline (PBS), fixed in 4% paraformaldehyde, and stained with 0.1% crystal violet. Hereafter, the number of colonies containing >50 cells was scored manually.

### 2.4. Apoptosis Detection

Annexin V-FITC/PI Apoptosis detection kit (556547, BD, California, USA) was applied to discriminate apoptotic cells. Briefly, cells were dispersed on 6-well plates (1.2 × 10^6^ cells/well) and then exposed to 0, 5, and 10 *μ*M of EO for 48 h. Subsequently, cells were collected and rinsed by PBS twice. Upon resuspending in the binding buffer (500 *μ*L), the cells were incubated at RT with Annexin V-FITC (5 *μ*L) and PI (10 *μ*L) in the darkness for 15 min. The measurement of the apoptotic cells was carried out by a flow cytometer (C6, BD, Franklin Lakes, USA).

### 2.5. Mitochondrial Membrane Potential (MMP) Detection

Flow cytometry was utilized to analyze the MMP with the help of JC-1 MMP detection kit (C2006, Biyuntian, Shanghai, China). JC-1 exhibits potential-dependent accumulation in the mitochondria, indicated by fluorescence emission shift from green (530 nm, FL1 channel) to red (590 nm, FL2 channel). After treating with 0, 5, and 10 *μ*M of EO for 48 h, cells were stained by JC-1 fluorophore at RT for 15 min. Next, the cells were rinsed by PBS and resuspended with the staining buffer. Finally, the MMP was detected by a flow cytometer.

### 2.6. Measurement of Intracellular Caspase-3

Next, intracellular caspase-3 was tested by flow cytometry. Briefly, cells were seeded on 24-well plates and cultured with 0, 5, and 10 *μ*M EO for 48 h. After that, the activation of caspase-3 was determined with a fluorogenic NucView™ 488 caspase-3 substrate for live cells based on operation instruction (30029-T, Biotium, California, USA). This substrate can permeate to cells with intact plasma membranes and detect the activation of caspase-3 in live cells. Finally, flow cytometry was employed to identify cells emitting green fluorescent signals (indicating the activation of caspase-3).

### 2.7. ROS Assay

The production of ROS was determined by flow cytometry. Upon treating with varied doses of EO in 6-well plates for 48 h, cells were cleaned and further cultured in a fresh medium with 10 *μ*M of dichloro-dihydro-fluorescein diacetate (DCFH-DA) for another 20 min as per operation instructions. In the next step, the cells were collected and rinsed, then, the fluorescence was determined by flow cytometry.

### 2.8. Quantitative RT-PCR (qRT-PCR)

The expressions of Bcl-2, Bax, PARP, and caspase-3 and -9 were detected at the mRNA level by qRT-PCR. In short, total RNA was isolated from cells treated with different doses of EO by TRIzol reagent (B511311, Sangon Biotech Co., Ltd., Shanghai, China). Next, RNA from each group was utilized for the synthesis of cDNA with RNA reverse-transcription kits (CW2569, CWBIO, Beijing, China) based on the manufacturer's protocol. Hereafter, SYBR Premix Ex TaqII (RR820A, Takara, Shiga, Japan) was employed to perform qRT-PCR. The results were normalized to the expression of GAPDH. The primer pairs of Bcl-2, Bax, PARP, caspase-3, caspase-9, and GAPDH are presented in Table 1.

### 2.9. Western Blotting

After treatment, cells were rinsed by PBS and lysed with radioimmunoprecipitation assay (RIPA) buffer. Then, the protein concentration was estimated by bicinchoninic acid (BCA) assay. Hereafter, the protein samples were separated by 5% SDS-polyacrylamide gel electrophoresis (SDS-PAGE) and transferred onto polyvinylidene difluoride (PVDF) membranes. Next, the membranes were blocked in 5% skimmed milk and immunoblotted overnight with primary antibodies against p-Akt (1 : 1000, AF0016), Akt (1 : 1000, AF6261), p-p38 (1 : 1000, AF4001), p38 (1 : 1000, AF6456), p-ERK (1 : 1000, AF1015), ERK (1 : 1000, 4695 s), c-Myc (1 : 1000, AF0358), and GADPH (1 : 5000, AF7021) at 4°C. Upon washing, the membranes were coincubated with HRP-conjugated secondary antibody for another 1 h at RT. At last, protein bands were assessed with the help of enhanced chemiluminescence (ECL) kits and quantified with ImageJ. All the primary antibodies were purchased from Affinity (Cincinnati, USA), except for ERK which is from Cell Signaling Technology (Massachusetts, USA).

### 2.10. Xenograft Tumor Growth Studies

Female BALB/c nude mice (6 weeks old, weighing 18-22 g) were supplied by Beijing Vital River Laboratory Animal Technology Co., Ltd. (Animal License No: SCXK Jing 2016-0011, Beijing, China). The mice were housed in specific pathogen-free conditions (50% humidity_,_23 ± 2°C, 12 h light/12 h dark cycle). All animal experiments were performed with the approval of the Animal Experimentation Ethics Committee of Zhejiang Eyong Pharmaceutical Research and Development Center (Certificate No. SYXK (Zhe) 2021-0033), and the experiments were conducted according to the guidelines of the Institutional Animal Care and Use Committee. For the generation of tumor xenografts, 2.5 × 10^6^ luciferase-labeled MDA-MB-231 and MDA-MB-453 cells were injected into the mammary fat pads of the mice. Once the tumor grew to 100 mm^3^, the animals were randomly assigned into 4 groups (*n* = 6): blank control (equal volume of saline), adriamycin (250 nM of adriamycin, D807083, Shanghai Macklin Biochemical Co., Ltd., Shanghai, China), EO low-dose (15 mg/kg/d of EO), and EO high-dose (30 mg/kg/d of EO) group. All the drugs were administered by intraperitoneal injection for 20 days. Tumor volume was calculated and recorded every 3 days for 21 days following the formula of volume = 0.5 × (length × width^2^). In addition, the growth and distribution of TNBC cells in the mammary fat pads of the mice were monitored by *in vivo* bioluminescence imaging system. At the end of the experiment, all mice were euthanized, and their tumors were resected and weighed immediately. The isolated tumors were fixed in paraformaldehyde solution (4%) and prepared as paraffin-embedded sections for hematoxylin-eosin (HE) staining, western blotting, and ELISA assay to observe the pathological changes, the expression of apoptosis-related proteins and Akt/p38 MAPK signaling pathway-associated proteins as well as the level of ROS.

### 2.11. Statistical Analysis

The data of the study were presented as mean ± SD and analyzed by SPSS 16.0. One-way ANOVA and the Tukey test were applied for multigroup comparison. The Kruskal-Wallis H test was applied if variances were not equal. A *p* < 0.05 was considered a statistically significant difference.

## 3. Results

### 3.1. EO Suppressed the Viability and Colony Formation of Human TNBC Cells

**Figure 1:**
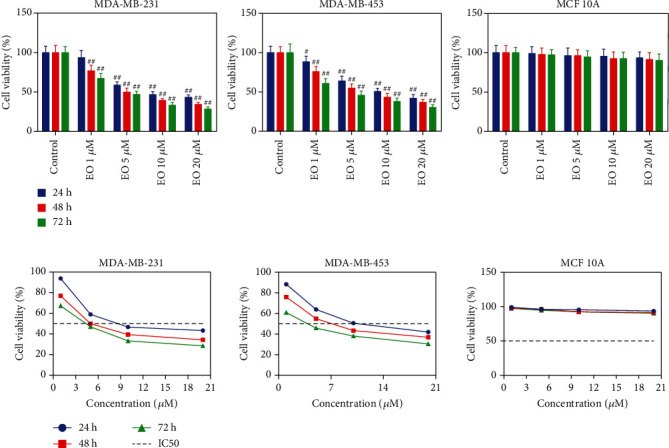
EO suppressed the viability of human TNBC cells. The viability of TNBC cell lines MDA-MB-231 and MDA-MB-453 and normal epithelial cell line MCF 10A treated with different concentrations of EO for 24 h, 48 h, and 72 h measured by MTT. Unless otherwise specified, all results were presented as mean ± SD. *n* = 3. EO, Eupalinolide O; TNBC, Triple-negative breast cancer; MTT, 3-(4,5-dimethylthiazol-2-yl)-2,5-diphenyltetrazolium bromide. ^#^*p* < 0.05, and ^##^*p* < 0.01 vs. Control, in all figures.

**Figure 2:**
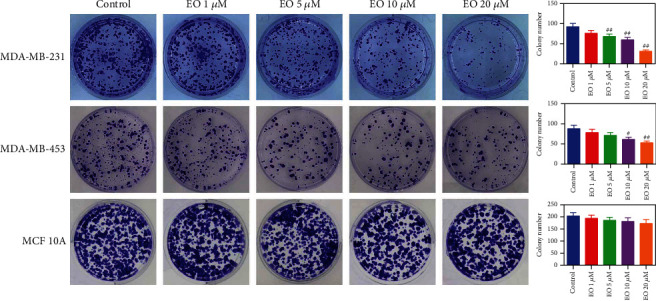
EO suppressed colony formation of human TNBC cells. The proliferation of MDA-MB-231, MDA-MB-453, and MCF 10A cells treated with different concentrations of EO detected by colony formation assay. *n* = 3.

To determine the cytotoxicity of EO, Human normal epithelial cell line (MCF 10A) and TNBC cell lines (MDA-MB-231 and MDA-MB-453) were cultured with 0, 1, 5, 10, and 20 *μ*M of EO for 24, 48, and 72 h, and then subjected to MTT assay. As exhibited in Figure 1, with the increasing time and concentration of EO, TNBC cells cultured with EO exhibited a sharp reduction in cell viability (*p* < 0.05). The IC_50_ value of EO for MDA-MB-231 cells at 24 h, 48 h, and 72 h was 10.34 *μ*M, 5.85 *μ*M, and 3.57 *μ*M, and for MDA-MB-453 cells was 11.47 *μ*M, 7.06 *μ*M, and 3.03 *μ*M, respectively. By contrast, MCF 10A cells appeared insensitive to EO treatment (*p* > 0.05). In fact, 5 and 10 *μ*M EO effectively repressed the viability of TNBC cells, and 20 *μ*M EO had a similar effect. Likewise, treatment with EO for 48 h obviously suppressed the growth of TNBC cells, and 72 h had a similar effect (*p* < 0.01). Based on this, 5 and 10 *μ*M of EO and 48 h were selected for the following experiments. Furthermore, the inhibitory effect of EO on TNBC cell proliferation was verified through colony formation assay. As depicted in Figure 2, EO suppressed the formation of TNBC cell colonies in a concentration-dependent manner. After exposure to 1, 5, 10, and 20 *μ*M of EO, the colony number of the MDA-MB-231 cells was reduced to 76.00 ± 7.00, 68.00 ± 6.08 (*p* < 0.01), 59.67 ± 6.11 (*p* < 0.01), and 31.33 ± 3.21 (*p* < 0.01), and the colony number of the MDA-MB-453 cells was decreased to 78.33 ± 8.08, 71.67 ± 6.66, 61.67 ± 5.13 (*p* < 0.05), and 53.00 ± 4.36 (*p* < 0.01). As expected, EO treatment had no remarkable impact on the colony formation of MCF 10A cells (*p* > 0.05).

### 3.2. EO Promoted the Apoptosis of Human TNBC Cells

**Figure 3:**
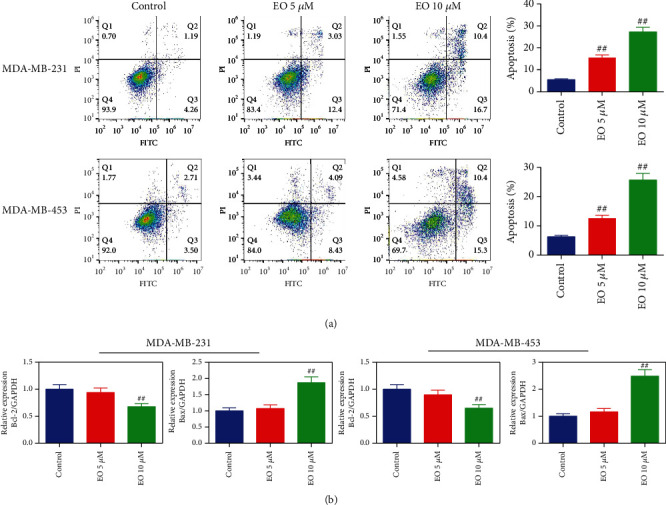
EO facilitated the apoptosis of human TNBC cells. (a) The apoptosis of TNBC cells evaluated by flow cytometry. (b) Bcl-2 and Bax mRNAs expression in TNBC cells tested by qRT-PCR. *n* = 3. qRT-PCR, quantitative RT-PCR.

Next, the role of EO in the induction of TNBC cell apoptosis was evaluated. The results of the apoptosis assay revealed that relative to the control cells, the apoptosis of TNBC cells was obviously increased upon EO treatment (*p* < 0.01, Figure 3(a)). To further confirm the impact of EO in the induction of apoptosis, the expression of Bax and Bcl-2 mRNA was detected. As displayed in Figure 3(b), Bcl-2 mRNA expression was evidently decreased while Bax mRNA expression was remarkably increased after TNBC cells were exposed to 10 *μ*M of EO (*p* < 0.01).

### 3.3. EO Induced Human TNBC Cells Apoptosis by the Intrinsic Apoptosis Pathway

**Figure 4:**
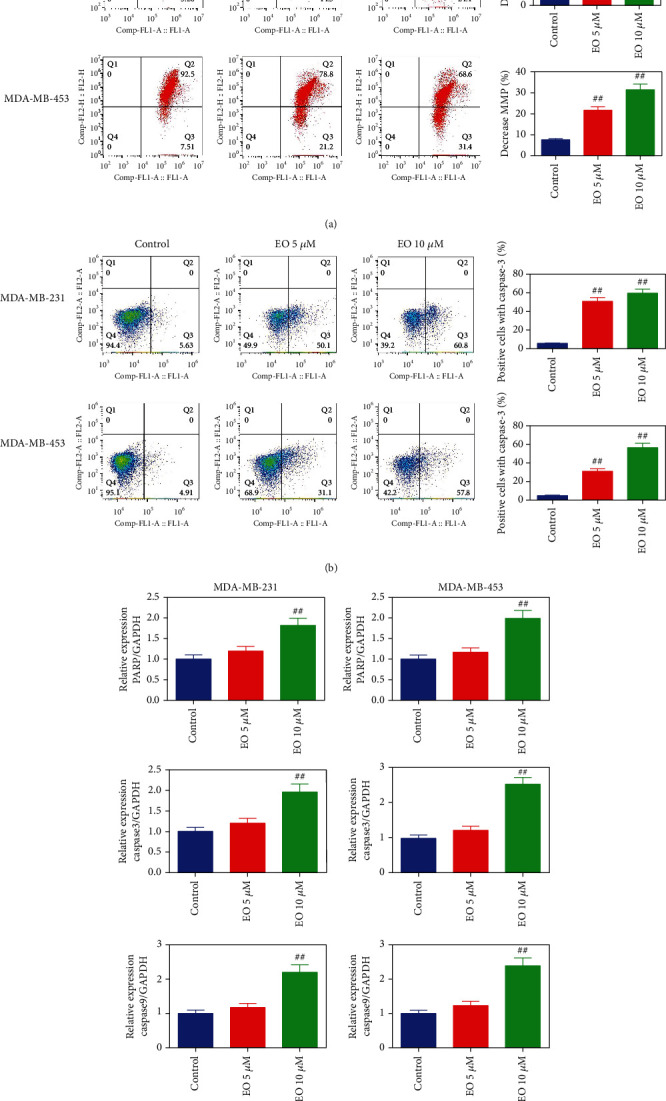
EO induced human TNBC cells apoptosis by intrinsic pathway. MMP loss (a) and caspase-3 expression (b) in TNBC cells evaluated by flow cytometry. (c) PARP and caspase-3 and -9 mRNA expression tested by qRT-PCR. *n* = 3. MMP, mitochondrial membrane potential.

To figure out whether mitochondrial-mediated intrinsic pathway participated in EO-triggered apoptosis, MMP loss, caspase-3 activity as well as the expression of PARP, and caspase-3 and -9 mRNAs were detected. As showcased in Figure 4(a), relative to the control group, the TNBC cells treated with 5 or 10 *μ*M EO had an obvious effect on the downregulation of MMP (*p* < 0.01). Consistently, results from flow cytometry and qRT-PCR also confirmed mitochondrial-mediated intrinsic pathway involved in EO-induced apoptosis for TNBC cells. As illustrated in Figure 4(b), the TNBC cells treated with EO, no matter in 5 *μ*M or 10 *μ*M, evidently enhanced the expression of caspase-3. Furthermore, qRT-PCR results demonstrated that the expression of PARP and caspase-3 as well as caspase-9 mRNAs in TNBC cells was significantly upregulated after treating with 10 *μ*M of EO (*p* < 0.01, Figure 4(c)).

### 3.4. EO Regulated ROS Generation and Akt/p38 MAPK Signaling Pathway in Human TNBC Cells

**Figure 5:**
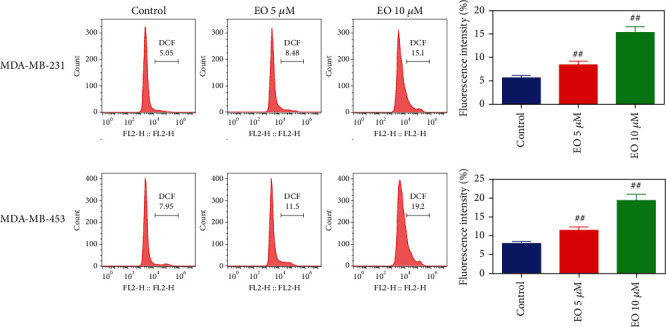
EO elevated the generation of ROS in human TNBC cells. ROS generation measured by flow cytometry. *n* = 3. ROS, reactive oxygen species.

**Figure 6:**
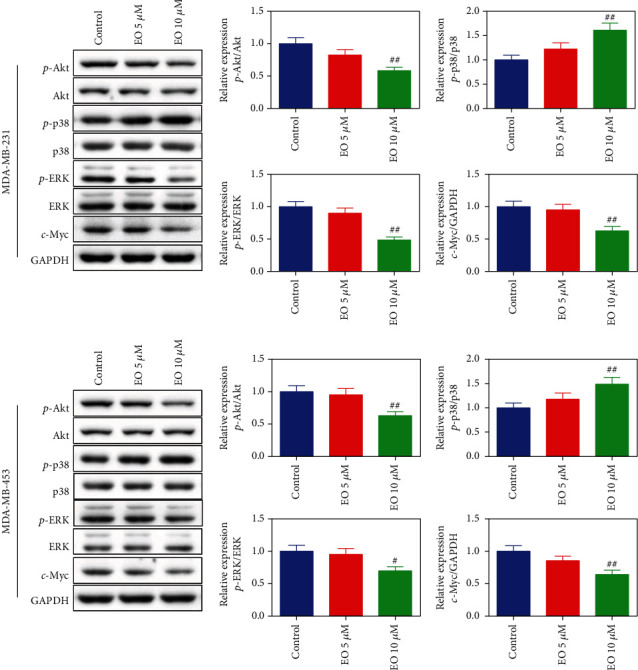
EO regulated Akt/p38 MAPK pathway-related proteins in human TNBC cells. c-Myc protein expression, Akt, p38, and ERK phosphorylation measured by western blotting. *n* = 3.

We explored if the effect of EO on proapoptosis in TNBC cells was mediated by ROS generation and Akt/p38 MAPK pathway. As described in Figure 5, after treating with 5 or 10 *μ*M EO, a dramatic upregulation of DCF fluorescence intensity was observed in the TNBC cells, which indicated ROS generation was significantly elevated (*p* < 0.01). Furthermore, the results of western blotting proved the phosphorylation of Akt and ERK as well as the expression of c-Myc protein were markedly decreased, while the phosphorylation of p38 was obviously increased after TNBC cells were treated with 10 *μ*M of EO (*p* < 0.05, Figure 6).

### 3.5. EO Inhibited the Growth of Tumors *In Vivo*

**Figure 7:**
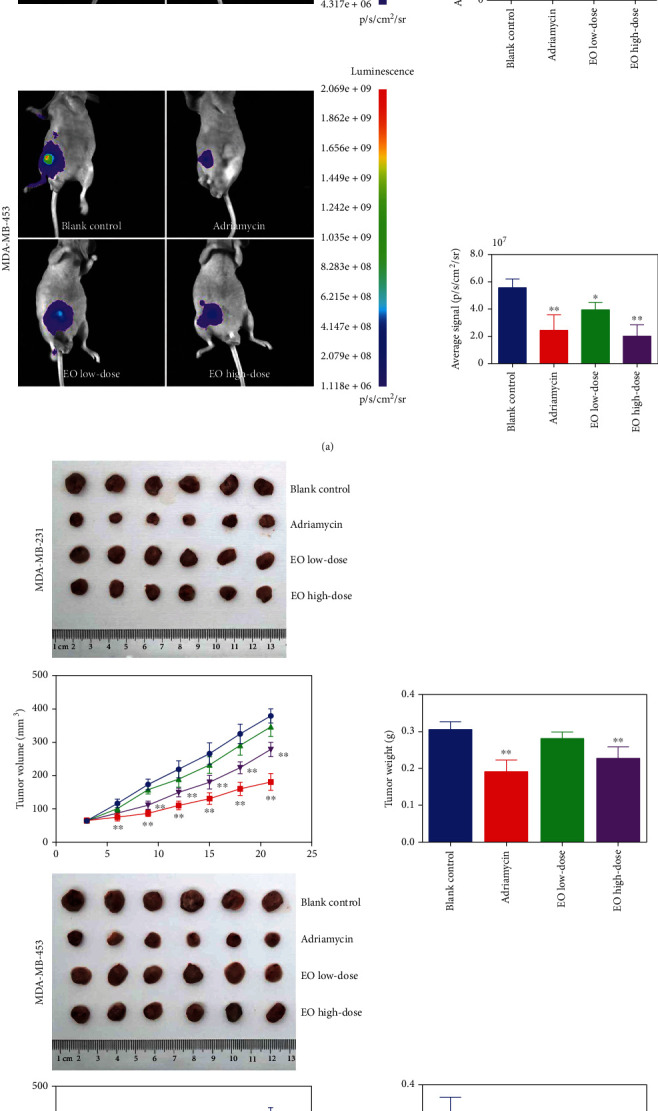
EO suppressed the growth of tumor in TNBC-tumor bearing mice. (a) The TNBC cell fluorescent intensity in tumor bearing mice detected by *in vivo* bioluminescence imaging system. (b) The tumor volume and tumor weight measured in tumor bearing mice. *n* = 6.

Subsequently, we verified the anticancer effect of EO *in vivo.* As exhibited in Figure 7(a), by *in vivo* bioluminescence imaging system, we observed the mice in the adriamycin group and EO high-dose group exhibited remarkably lower fluorescent intensity than that of the blank control group, regardless of whether the mice were injected with MDA-MB-231 or MDA-MB-453 cells (*p* < 0.01). In addition, low dose of EO obviously weakened the fluorescent intensity of mice injected with MDA-MB-453 cells (*p* < 0.05). Similarly, whether the mice were injected with MDA-MB-231 or MDA-MB-453 cells, there was an apparent decrease of tumor volume in mice of the adriamycin group and EO high-dose group from day 6 to day 9 relative to the blank control group (*p* < 0.01). Meanwhile, low dose of EO effectively decreased the tumor volume of mice injected with MDA-MB-453 cells from day 12 (*p* < 0.05). Furthermore, at the end of the experiment, we observed the average tumor weight of the adriamycin group and EO high-dose group was lower than that of the blank control group, regardless of whether the mice were injected with MDA-MB-231 or MDA-MB-453 cells (*p* < 0.01, Figure 7(b)). Furthermore, low dose of EO evidently reduced the tumor weight of mice injected with MDA-MB-453 cells (*p* < 0.05).

**Figure 8:**
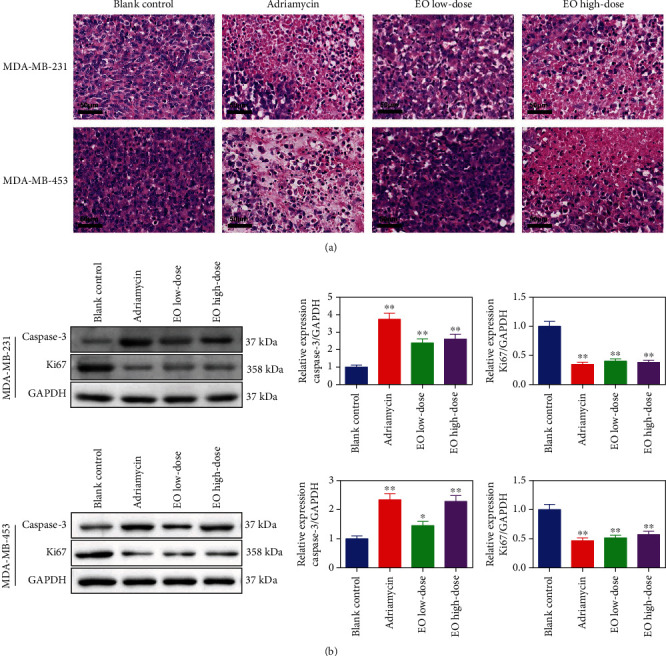
EO destroyed the structure of the tumor tissues and modulated caspase-3 and Ki67 expression in TNBC-tumor bearing mice. (a) The pathological changes of the tumor tissues observed by HE staining, magnification ×400, scale bar = 100 *μ*M. (b) Caspase-3 and Ki67 expression of the tumor tissues measured by western blotting. *n* = 3.

We also observed the tumor tissues by HE staining and found that compared to the blank control group the tumor cells were slightly enlarged, the number of heterotypic cells decreased, the structure of the tumor tissues destroyed, some cells exhibited obvious pyknosis, and necrosis in addition to massive leukocyte and lymphocyte infiltration in the adriamycin group, EO low-dose group, and EO high-dose group, indicating the growth of tumor cells was suppressed (Figure 8(a)). In addition, the results of western blotting revealed that upon treatment with low-dose EO, high-dose EO, or adriamycin, caspase-3 protein expression was increased while the Ki-67 protein expression was suppressed in the TNBC-tumor bearing mice (whether the mice were injected with MDA-MB-231 or MDA-MB-453 cells) compared with the mice in the blank control group (*p* < 0.05, Figure 8(b)).

### 3.6. EO Regulated the Generation of ROS and the Expression of Akt/p38 MAPK Signaling Pathway-Associated Proteins *In Vivo*

**Figure 9:**
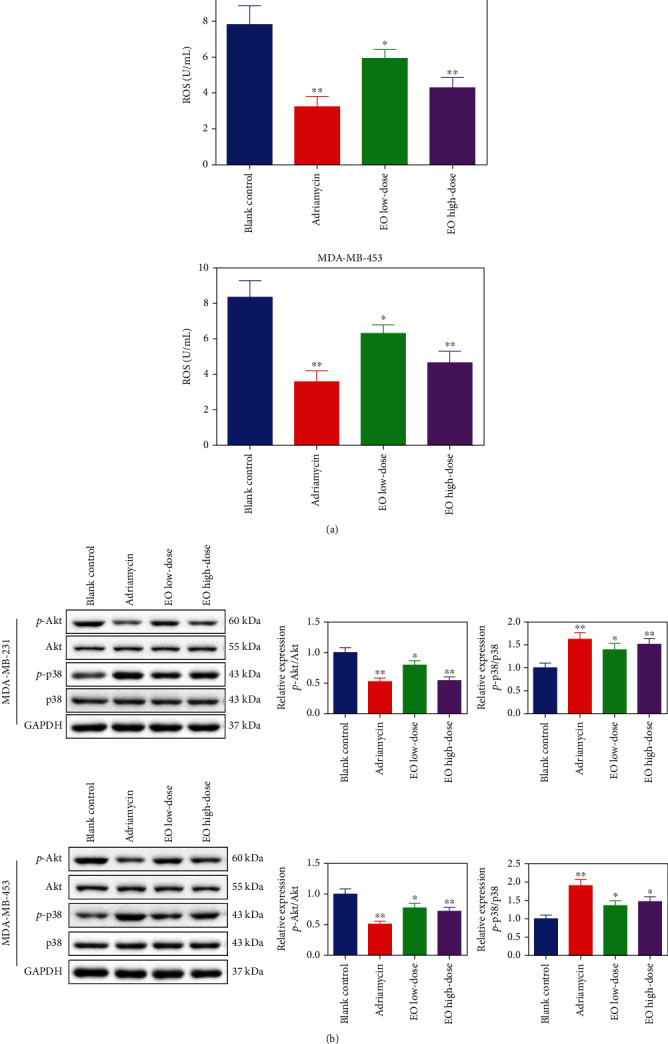
EO regulated ROS level and Akt/p38 MAPK signaling pathway-associated proteins in TNBC-tumor bearing mice. (a) The level of ROS detected by ELISA assay. (b) Akt and p38 phosphorylation measured by western blotting. *n* = 3.

Finally, we further confirmed the mechanism of anticancer action of EO in the TNBC-tumor bearing mice. As showcased in Figure 9, the results of the ELISA assay revealed the level of ROS in the adriamycin group and EO groups was lower than that of the blank control group, regardless of whether the mice were injected with MDA-MB-231 or MDA-MB-453 cells (*p* < 0.05). Likewise, whether the mice were injected with MDA-MB-231 or MDA-MB-453 cells, by western blotting, we observed Akt phosphorylation was significantly downregulated while p38 phosphorylation was obviously upregulated upon treatment with low-dose EO, high-dose EO, or adriamycin in the TNBC-tumor bearing mice (*p* < 0.05).

## 4. Discussion

Natural products serve a prominent role in the field of novel drug research and development due to their abundant resources, few side effects, and variable bioavailability [16]. A large number of studies have been conducted to study the function and structure of natural products since the 1850s, which have promoted the knowledge of their anticancer properties [17]. Recently, *Eupatorium lindleyanum* DC. has been reported to have a series of biological activities, such as antioxidant [18]and anticancer [14] as well as anti-inflammatory [19]. Meanwhile, Zhang et al. have reported that Eupalinolide B, a kind of compound extracted from *Eupatorium lindleyanum* DC., inhibited hepatic carcinoma via inducing ferroptosis and the ROS-ER-JNK pathway [20]. Furthermore, some published studies have reported that Eupalinolide J can not only induce apoptosis, MMP disruption, cell cycle arrest, and DNA damage in TNBC cells but also inhibits the growth of TNBC by targeting the STAT3 signaling pathway [3, 21]. However, the effect and mechanism of EO (another compound extract from *Eupatorium lindleyanum* DC.) in TNBC are still unknown. In the present study, we validated that EO induces TNBC cell apoptosis by modulating ROS generation and Akt/p38 MAPK pathway.

As is well known, cancer develops from the clonal evolution of a single cell, thus, suppressing the cloning formation of tumor cells is essential for treating TNBC [22]. In addition, the induction of apoptosis is the major mechanism for numerous anticancer drugs to treat cancers [23]. Generally, there are two pathways involved in apoptosis, specifically, the receptor-mediated extrinsic pathway and the mitochondrial-mediated intrinsic pathway [24]. For the intrinsic pathway, Bcl-2 family members, such as Bax and Bad (proapoptotic factors) as well as Bcl-xl and Bcl-2 (antiapoptotic factors), are essential in the induction of apoptosis [25]. In response to proapoptotic signals, the ratio of Bcl-2/Bax expression will be reduced and the MMP will be disrupted followed by the release of cytochrome c and activation of caspases. Ultimately, cell apoptosis takes place [26]. As per the previous report, some drugs inhibit the development of TNBC by inhibiting clone formation [27] and inducing apoptosis of TNBC cells [28]. Meanwhile, promoting clone formation [29] and inhibiting cellular apoptosis for TNBC cells will facilitate the development of TNBC [30]. Furthermore, some studies have demonstrated that compounds isolated from *Eupatorium lindleyanum* DC. can inhibit the progression of cancer by induction of cell apoptosis, cycle arrest, and autophagy [14]. Consistently, in this study, EO repressed the clone formation and induced the apoptosis of TNBC cells. At the same time, destruction of MMP, blunt Bcl-2 mRNA expression, enhanced Bax mRNA expression, and activated caspase-3 and -9 were evidently observed in EO-treated TNBC cells, which offered direct evidence that EO could induce the apoptosis of TNBC cells by mitochondrial-mediated intrinsic pathway.

MAPK family is composed of p38 MAPK, JNK, and ERK, which are crucial in cellular stress responses [31]. As originally known, ERK activation is vital for cell proliferation, while p38 activation and JNK activation are generally responsible for the modulation of apoptosis [32]. In addition, Akt facilitates cell survival by suppressing apoptosis [33]. Based on the previous report, Bcl-2 and Bcl-xl have been verified as direct targets of the Akt/p38MAPK pathway [34]. Modulation of the Akt/p38MAPK pathway will lead to the regulation of Bcl-2 expression and Bcl-xl expression [35]. In the present study, we observed that the phosphorylation of p38 was elevated, while the phosphorylation of ERK and Akt was reduced upon EO treatment, which were in line with the recent report on the function of the Akt/p38 MAPK pathway in cancer [36, 37]. Some studies have also proved that inhibiting ERK activity can induce cell death through both intrinsic and extrinsic apoptotic pathways [38]. It has been reported that some anticancer agent-induced apoptosis was associated with the inhibition of ERK activation [39].

As is well known, ROS is a kind of by-product of cellular metabolism, which contributes to many vital biological processes, including autophagy and apoptosis as well as cell cycle arrest in various human cancer cell lines [40, 41]. Recently, a study conducted by Zhang et al. has demonstrated that ROS can stimulate Akt/p38 MAPK pathway directly *in vitro*, thereby mediating cell cycle arrest and apoptosis in human hepatoma cells [17]. Besides, it is reported that the elevated ROS can inhibit Bcl-2 expression and activate Bax expression in the mitochondria [42]. F1012-2, another compound isolated from *Eupatorium lindleyanum* DC., has been reported to cause DNA damage in TNBC cells by increasing ROS production [43]. In this study, the generation of ROS was notably elevated in TNBC cells upon exposure to EO. Our results implicated that EO-induced apoptosis in human TNBC cells was related to ROS generation and Akt/p38 MAPK signaling pathway. However, whether there was a relationship between ROS generation and the Akt/p38 MAPK pathway under the context of EO treatment was not explored. Further experiments are needed to research the relevant mechanisms.

We also verified the anticancer effects of EO *in vivo* by performing a xenograft tumor study. Tumor volume and weight, as well as TNBC cell fluorescent intensity, were reduced under EO treatment. In addition, HE staining and western blotting indicated that EO not only effectively destroyed the structure of the tumor tissues but also upregulated caspase-3 protein expression and suppressed Ki-67 protein expression. This result is in line with previous research on the anti-TNBC agents in mice. The researchers gave TNBC mice the corresponding treatment and then detected the expression of caspase-3 as well as Ki67. They found that anti-TNBC agents could enhance the expression of caspase-3 and decrease the expression of Ki67 to induce TNBC cell death [44, 45]. Moreover, *in vivo* experiments also revealed Akt phosphorylation and ROS level were downregulated while p38 phosphorylation was upregulated upon treatment with EO in TNBC-tumor bearing mice, which further confirmed the finding of the *in vitro* experiments.

In summary, our results revealed the function and mechanism of EO in TNBC. Specifically, EO possesses an anti-TNBC ability both *in vivo* and *in vitro*, and the possible mechanism may be associated with the induction of apoptosis regulated by ROS generation and the Akt/p38MAPK signaling pathway. Our results suggested that EO could serve as a new agent for therapy against TNBC.

## Figures and Tables

**Table 1:** qRT-PCR primers.

Gene	Forward primer	Reverse primer
Human Bcl-2	GTCTTCGCTGCGGAGATCAT	CATTCCGATATACGCTGGGAC
Human Bax	CATATAACCCCGTCAACGCAG	GCAGCCGCCACAAACATAC
Human PARP	TGCAATGGTCGTGAACAACCT	CAACTGGGACCGTTGAAACTG
Human caspase-3	CATGGAAGCGAATCAATGGACT	CTGTACCAGACCGAGATGTCA
Human caspase-9	ATGTCGGACTACGAGAACGAT	TGATGCGTGAGGGGTCGAT
Human GAPDH	GGAGCGAGATCCCTCCAAAAT	GGCTGTTGTCATACTTCTCATGG

## Data Availability

The data used to support the findings of this study are available from the corresponding authors upon request.
